# The Efficacy of High-Volume Evacuators and Extraoral Vacuum Aspirators in Reducing Aerosol and Droplet in Ultrasonic Scaling Procedures during the COVID-19 Pandemic

**DOI:** 10.1055/s-0041-1739448

**Published:** 2022-01-11

**Authors:** Trijani Suwandi, Vidya Nursolihati, Mikha Sundjojo, Armelia Sari Widyarman

**Affiliations:** 1 Department of Periodontics, Faculty of Dentistry, Trisakti University, Grogol Jakarta Barat, Indonesia; 2 Department of Microbiology, Faculty of Dentistry, Trisakti University, Grogol Jakarta Barat, Indonesia

**Keywords:** aerosol, droplet, extraoral vacuum aspirators, high-volume evacuators, ultrasonic scaling

## Abstract

**Objective** SARS-CoV-2 can be carried by aerosols and droplets produced during dental procedures, particularly by the use of high-speed handpieces, air-water syringes, and ultrasonic scalers. High-volume evacuators (HVEs) and extraoral vacuum aspirators (EOVAs) reduce such particles. However, there is limited data on their efficacy. This study aimed to determine the efficacy of HVE and EOVA in reducing aerosols and droplets during ultrasonic scaling procedures.

**Materials and Methods** Three ultrasonic scaling simulations were conducted on mannequins: 1. saliva ejector (SE) was used alone (control); 2. SE was used in combination with HVE; and 3. SE was used in combination with HVE and EOVA. Paper filters were placed on the operator's and assistant's face shields and bodies, and the contamination of aerosols and droplets was measured by counting blue spots on the paper filters.

**Statistical Analysis** All data were analyzed for normality using the Kolmogorov–Smirnov test. The differences between each method were analyzed using a two-way ANOVA, followed by a posthoc test. The differences were considered statistically significant when *p* < 0.05.

**Result** Using HVE and EOVA reduced aerosols and droplets better than using SE alone or SE and HVE: the posthoc test for contamination revealed a significant difference ( *p* < 0.01). The assistant was subjected to greater contamination than the operator during all three ultrasonic scaling procedures.

**Conclusion** The usage of HVE and EOVA significantly reduced aerosols and droplets compared with using SE solely. Using these techniques together could prevent the transmission of airborne disease during dental cleanings, especially COVID-19. Further studies of aerosol-reducing devices are still needed to ensure the safety of dental workers and patients.

## Introduction

Coronavirus disease 2019 (COVID-19) is an infectious respiratory disease caused by severe acute respiratory syndrome Coronavirus 2 (SARS-CoV-2). 1 It has been declared as a pandemic by the World Health Organization (WHO) and millions of cases continue to be reported around the world. Until now, several mutations and variants of SARS-CoV-2 have emerged throughout the world, namely, B.1.1.7 (alpha), B.1.351 (beta), P1 (gamma), and B.1.617.2 (delta). 2 Recent studies suggested that delta variant spreads faster, causes more infections, is 40 to 60% more contagious than the alpha variant, and may be the most transmissible variant. 2 3

SARS-CoV-2 is mainly transmitted between people through contact and respiratory droplets routes. 4 5 Respiratory droplets, which are > 5 to 10 μm in diameter, are released when a person coughs, sneezes, or talks, while droplets ≤ 5 μm in diameter are referred to as droplet nuclei or aerosols, which can remain in the air over long distances and time. 4 An additional category of larger droplets, more than 50 μm in diameter, is described as splatter in some studies. 6 7 Even though COVID-19 transmission through contact or droplets inhalation is considered as the main route of transmission, another potential route of transmission can occur via airborne through inhalation of aerosol and droplet exhaled by an infected person. 7 8

Dental procedures that generate aerosols (“aerosol-generating procedures”) are considered as high-risk mode of SARS-CoV-2 airborne transmission. 4 9 Several reports indicate that ultrasonic scaling procedures are one of the largest major sources of aerosols and droplets 10 that are mainly contaminated with bacteria and viruses. 11 Therefore, safety and infection control procedures are very important to minimize the risk of transmission, one of which is by reducing the number of aerosols and droplets. 10 The currently used aerosol-reducing devices are low-volume evacuators such as saliva ejectors (SEs), high-volume evacuators (HVEs), and extraoral vacuum aspirators (EOVAs). 5 10 12 13

The SEs usually used by dentists have small suction tips that are not large enough to remove a large number of aerosols. 10 The Centers for Disease Control and Prevention (CDC) recommend aerosols contamination control during dental treatment using techniques and devices such as high-velocity air evacuation and HVEs and EOVAs. It could reduce aerosols up to 93 to 96% in dental clinics. 10 12 13 However, the research methods and data discussing on the effectiveness of using these devices to reduce aerosols and droplets during ultrasonic scaling are scarce. Moreover, no study in the literature has compared the efficacy of HVE and EOVA in reducing aerosols when used alone or together. This is the first study that compares the effectiveness of SE alone, SE and HVE, as well as SE, HVE and EOVA in reducing aerosols and droplets in dental procedure (ultrasonic scaling) both on operators and dental assistants using disclosing solutions and paper filters to capture aerosol and droplet dispersion.

## Materials and Methods

**Fig. 1:**
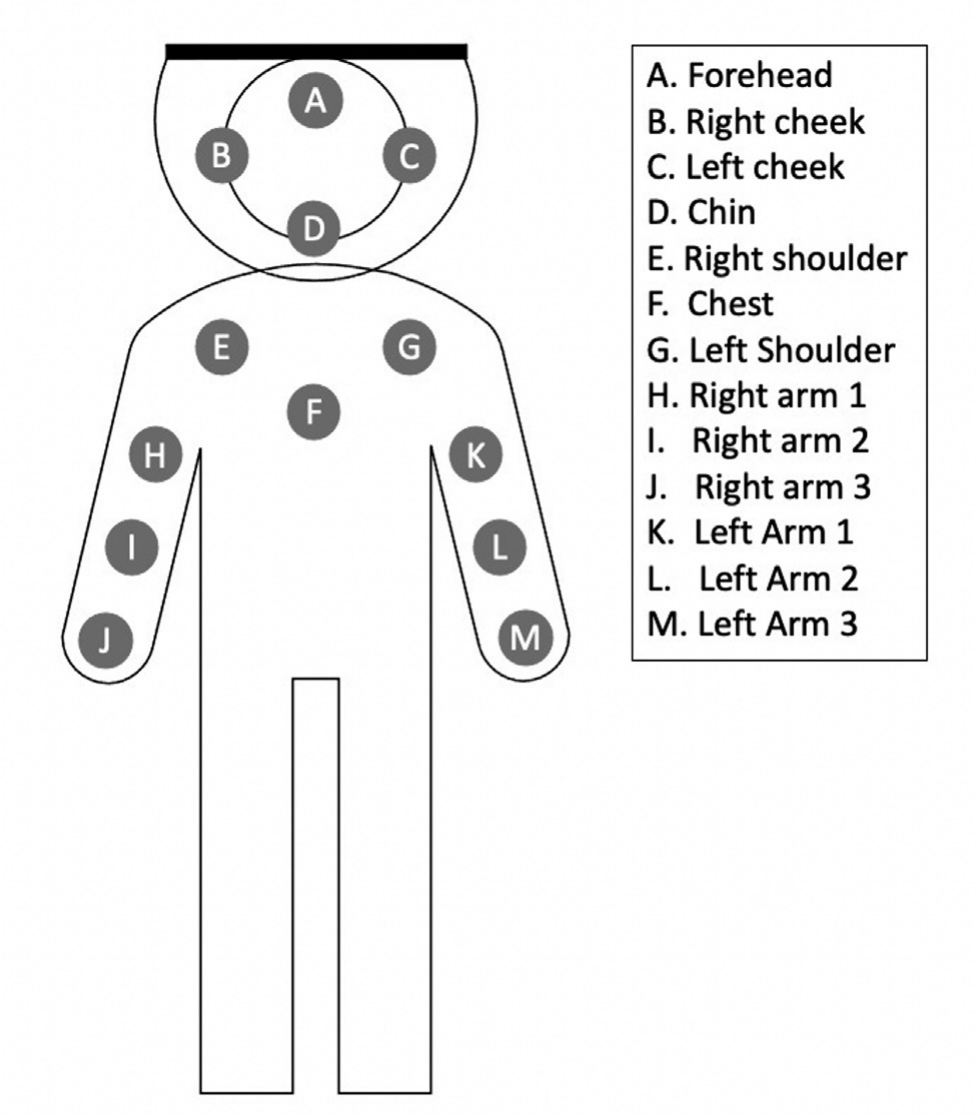
Schematic distribution of the paper filters on the body (A = forehead; B = right cheek; C = left cheek, D = chin; E = right shoulder; F = chest, G = left shoulder; H = right arm 1; I = right arm 2; J = right arm 2; K = left arm 1; L = left arm 2; M = left arm 3).

The method used in this study is a modification from a pilot study by Veena et al. 14 Ultrasonic scaling simulation was performed using a piezoelectric ultrasonic scaler (EMS, Swiss) on a mannequin with jaw simulator (Kavo Dental, Germany) and placed in supine position on dental unit (Clesta II, Belmont, Japan). The dental unit and ultrasonic scaler water lines was filled with a mixture of 10 mg disclosing solution (GC Tri Plaque ID Gel, Japan) and 1000 mL aquadest. Paper filter with grids (Whatman ME25/21ST, Sigma-Aldrich, St. Louis, Missouri) made from mixed cellulose ester membranes (cellulose acetate and cellulose nitrate) was used to capture aerosol and droplet, because it has uniform microporous structure, which gives high-flow rates for greater adsorption. The paper filter with grid lines provide excellent contrast for easier particle detection to assist in manual counting procedures. These paper filters were placed with adhesive tapes on the operator's and assistant's face shields (which covered the forehead, both cheeks, and chin) and on several parts of the bodies: chest, right shoulder and left shoulder, and three paper filters on both arms with 15 cm distance on each arm. Each paper filter was marked with code, according to their position, as shown in Fig. 1 .

### Ultrasonic Scaling Simulation

The operator performed the scaling simulation at 11 o'clock position, while the assistant held the SE or HVE positioned at 1 o'clock position in manner of mannequin head considered as 12 o'clock position. Each simulation was performed for 15 minutes. Based on the aerosol-reducing devices used in the procedures, the scaling simulations were divided into three groups: group 1, the control group, using SE alone; group 2 using SE and HVE; and group 3 using SE, HVE, and EOVA (Coxo, Guangdong, China) together where the suction hood distanced 15 cm from mannequin mouth. The scaling procedure was repeated 20 times for each group.

### Measurement of Aerosol and Droplet Contamination

**Fig. 2:**
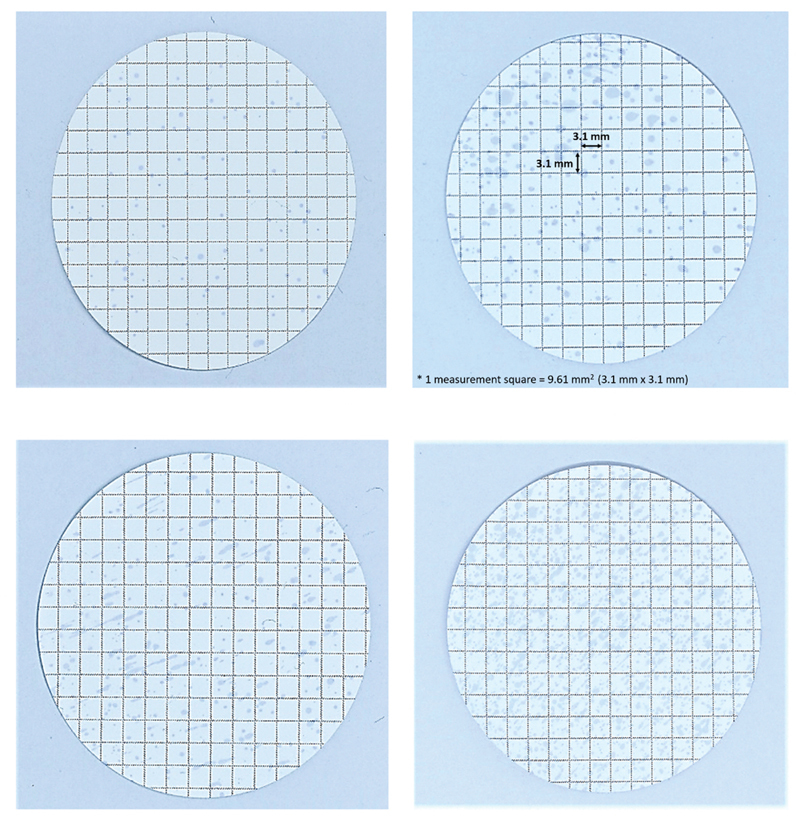
Contamination visible on paper filters.

The extent aerosols and droplets contamination on the paper filters was measured in mm ^2^ units. Square on paper filter was categorized as contaminated if there is at least a blue spot inside the square. Measurement was done by manually counting total contaminated squares on paper filters multiplied by 9.61 mm ^2^ (square area of each square on paper filter). The total contaminated area on each paper filter was measured by counting the number of contaminated squares. The mean contaminant area is the mean contaminated area on paper filters from 20 repeated scaling simulations of each group ( Fig. 2 ).

### Statistical Analysis

All data was collected and analyzed for normality using the Kolmogorov–Smirnov test. The differences between each method were analyzed using a two-way ANOVA, followed by a posthoc test. The differences were considered statistically significant when *p* < 0.05 (SPSS statistic version 20, IBM, USA).

## Results

**Fig. 3:**
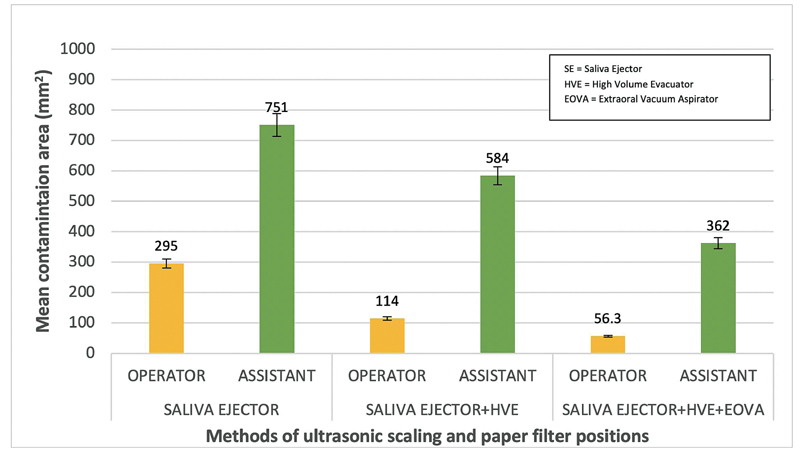
Means of the contamination area according to scaling groups and paper filter positions. EOVA, extraoral vacuum aspirator; HVE, high-volume evacuator; SE, saliva ejector.

The mean contamination areas found on the operator and assistant following the three methods of ultrasonic scaling are shown in Fig. 3 . Of the three methods, ultrasonic scaling using SE, HVE, and EOVA (group 3) was most effective at reducing aerosols.

Statistical analysis using a two-way ANOVA test showed that the ultrasonic scaling procedure using SE, HVE, and EOVA significantly reduced aerosols and droplets ( *p* < 0.01) compared with using SE alone and SE and HVE ( Table 1 ).The results of the posthoc test for contamination areas on the operator and assistant showed a significant difference ( *p* < 0.01), as the assistant was subjected to more contamination than the operator during the ultrasonic scaling methods used in all three procedures ( Table 2 ).

**Table 1:** Statistical analysis of contamination area from each method used in ultrasonic scaling procedures using a two-way ANOVA test

Comparison						
Method	Method	Mean difference	SE	df	t	*p* -Value
SE	SE + HVE	179	7.42	1456	24.1	< 0.001
	SE + HVE + EOVA	314	7.33	1456	42.8	< 0.001
SE + HVE	SE + HVE + EOVA	135	7.42	1456	18.2	< 0.001

Abbreviations: EOVA, extraoral vacuum aspirator; HVE, high-volume evacuator; SE, saliva ejector.

**Table 2:** Results of the posthoc test for contamination present on the operator and assistant following the ultrasonic scaling procedures

Comparison						
Position	Position	Mean difference	SE	df	t	*P* -Value
Operator	Assistant	– 408	6.03	1456	–67.6	< 0.001

## Discussion

This study shows that using HVE and EOVA in ultrasonic scaling procedures reduces aerosols and droplets contamination on paper filters around the working zone ( Fig. 3 ). Previous studies have shown that SE was not effective enough to reduce aerosols production in ultrasonic scaling procedures. It only reduced water from the floor of the mouth but continued to spread aerosols throughout the working area, including the dental operator's and assistant's personal workspace. 14 15

SARS-CoV-2 can transmit and infect people through aerosol transmission, 16 thus having HVE at constant operation during dental operation can significantly reduce aerosol production up to 90 to 98%. 17 This study presented significantly lower mean contamination area when using HVE combined with SE. These findings ultimately lead to the importance and the high-necessity of having HVE in dental settings, especially during this pandemic era when dental practices are needed. Moreover, to further reduce possible aerosol contamination use EOVA with HVE. Our study also showed a further mean reduction in the contamination area after using EOVA with HVE. A previous study conducted by Shahdad et al particularly documented the efficacy of splatter contamination reduction in a dental aerosol-generating procedure by using EOVA. 18

The utilization of EOVA was uncommon in prepandemic situation, but its efficacy in reducing aerosols has been assessed. 19 Since the pandemic, every measure to potentially reduce aerosol transmission in dental practices is sought out, with hopes that dental practitioners can still continue their work during the pandemic. 20 With the result from this study, combination usage of HVE with EOVA becomes prominent for dental settings.

The two-way ANOVA test revealed less droplets were captured on the paper filters when HVE and EOVA were used ( Table 1 ). HVE requires a dental assistant to hold and direct the tip while maintaining a 6 to 15 mm distance from the working zone. 5 17 EOVA is more effective when located 14 mm from the working zone rather than 18 mm. 21 However, studies on the effectiveness of HVE and EOVA are still limited and further studies on the design, power, and recommended instructions for using these devices are still needed.

The extent and contamination of droplets and aerosols on person (operator, patient, and assistant) also need to be identified to manage the risk of disease transmission. Although droplets contamination on person during ultrasonic scaling procedure have been reported in many papers, only few papers considered at contamination of the assistant, of which were found on assistant's head and chest. 6 Our study result shows more contamination in the assistant's body compared with the operator's body ( Table 2 ). This variance between papers possibly results from different methodologies used in each paper.

Aerosols produced by ultrasonic scaling can remain in the air for 30 minutes. 14 Studies also suggest that SARS-CoV-2 can persist and remain alive in aerosols for hours, 22 thus it is recommended that dental workers do not immediately remove their personal protective equipment (PPE) after work. 14 Reducing the risk of airborne contamination from ultrasonic scaling procedures could be achieved by using HVE, but using SE alone is not recommended. 23 Alternatively, minimizing the risk of infection from airborne particles in dental treatment facilities could be achieved by several means, aside from using the combination of SE, HVE, and EOVA, such as; using filter in the ventilation systems (for example, high-efficiency particulate air [HEPA] filters), 24 using ultraviolet (UV) light for sterilization prior to dental service, 25 and/or applying negative pressure during dental procedure. By applying negative pressure, airborne time of aerosols becomes limited as the aerosols were drawn downward, thus reducing aerosol contaminants further. 26 Furthermore, the risk of microbial transmission could be minimized by decreasing microbial bioload with preprocedural mouth rinse and dental unit water line decontamination. 27

This study results show that dental workers can benefit from the utilization of HVE and EOVA during ultrasonic scaling, as it reduced significant amount of aerosols and droplets around patient's mouth, thereby decreasing the risk of cross-contamination of pathogens. The advantages of the method used in the study are it is a simple and low-cost method, easy to set-up, and also reproducible. The disadvantages are the range areas of aerosol and droplet capture are limited. Our study still has several limitations. It only counts aerosols and droplets contamination on the operator's and assistant's bodies, and there is no description on the pathogens composition that serve as the source of infection. We suggest future study on aerosol contamination in larger areas with microbiological evaluation.

## Conclusion

This study findings showed that using HVE and EOVA with SE reduced the aerosols and droplets production during ultrasonic scaling procedures significantly compared with using SE solely. These devices could be a beneficial addition to dentistry practices, in order to reduce the risk of disease transmission, especially COVID-19. The results also indicate that dental assistants are at greater risk for being contaminated by aerosols and droplets. Therefore, the use of complete PPE is crucial. Further studies of aerosol-reducing devices are still needed to ensure the safety of dental workers and patients.
